# Supplementary material for: Causal interplay between lactose intolerance and gut microbiota: a combined bidirectional Mendelian randomization and in vivo validation study
Source: Front Nutr. 2026 Jun 1;13:1803337. doi: 10.3389/fnut.2026.1803337 (PMC13265576; doi:10.3389/fnut.2026.1803337)
Supplement: Supplementary file 1 [file Data_Sheet_1.zip › supplementary materials/Forward/forest plot/ebi-a-GCST90027828.finngen_R12_E4_LACTONAS.pdf]

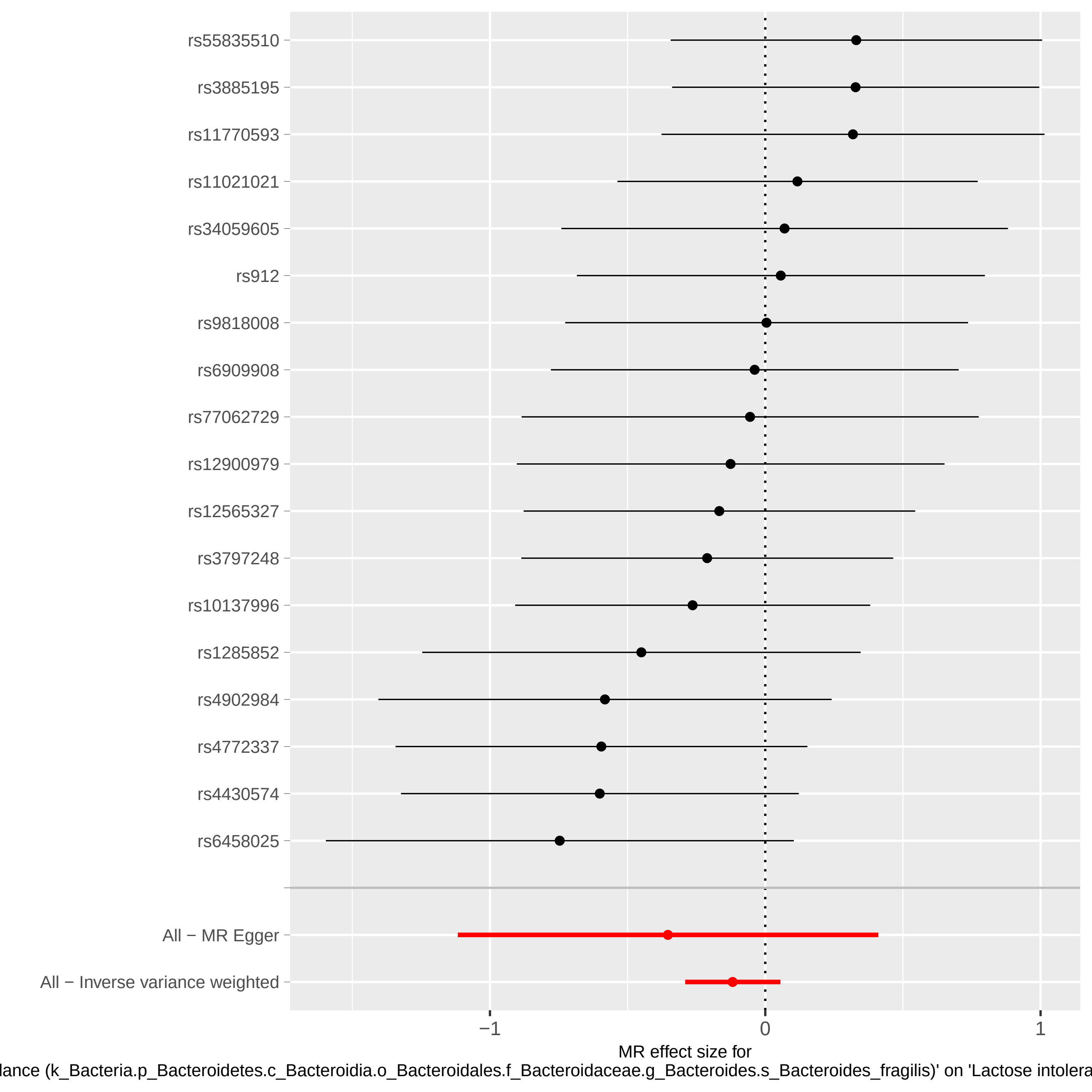
rs55835510
rs3885195
rs11770593
rs11021021
rs34059605
rs912
rs9818008
rs6909908
rs77062729
rs12900979
rs12565327
rs3797248
rs10137996
rs1285852
rs4902984
rs4772337
rs4430574
rs6458025
All − MR Egger
All − Inverse variance weighted
−1
0
1
MR effect size for
lance (k_Bacteria.p_Bacteroidetes.c_Bacteroidia.o_Bacteroidales.f_Bacteroidaceae.g_Bacteroides.s_Bacteroides_fragilis)' on 'Lactose intolera
